# Responses of *Littorina* spp. Intertidal Snails to Thermal Extremes Indicate Countergradient Variation in Fitness

**DOI:** 10.1002/ece3.70926

**Published:** 2025-01-29

**Authors:** Ffion Dylan Titmuss, Molly A. Albecker, Katie E. Lotterhos

**Affiliations:** ^1^ Department of Applied Ocean Physics and Engineering Woods Hole Oceanographic Institution Woods Hole Massachusetts USA; ^2^ Department of Biology and Biochemistry University of Houston Houston Texas USA; ^3^ Department of Marine and Environmental Sciences Northeastern University Nahant Massachusetts USA

**Keywords:** countergradient variation, latitudinal gradient, local adaptation, marine, thermal tolerance

## Abstract

Global change models predict not only a steady increase in temperatures but also an increase in the occurrence of hot and cold extremes. Organisms' responses to thermal extremes will depend on species‐specific traits and the degree of within‐species variation (among populations), with populations from warmer latitudes often predicted to have higher thermal tolerance than populations from colder latitudes. The evolution of population‐specific responses, however, can be limited by gene flow that homogenises populations. Here, we investigate this relationship with a study of the survival of *Littorina littorea* , *L. obtusata* , and *L. saxatilis* —marine snails with varying dispersal potential—collected on either side of a known biogeographic break. Snails were laboratory‐acclimated for several weeks before undergoing exposures to extreme heat, extreme cold, or ambient conditions, and individual mortality was recorded after each exposure. In line with common predictions, we observed that the degree of population divergence in survival under thermal extremes was negatively related to dispersal potential, and that populations from the colder latitude generally had higher survival of sub‐freezing temperatures. Contrary to common predictions, however, we observed greater survival after extreme heat in populations from colder latitudes than in their warmer‐latitude counterparts, a pattern known as countergradient variation. This experiment highlights counterintuitive responses to thermal extremes, emphasising that colder‐latitude populations could experience population growth under more extreme climates due to higher survival at both hot and sub‐freezing thermal extremes.

## Graphical abstract

Comparing six marine snail populations across three congeneric species, we identified varying differentiation both among species and among populations within species in their responses to thermal extremes. The degree of population divergence in their mortality was negatively related to species‐level dispersal potential, and populations from the colder latitude generally had higher survival of sub‐freezing temperatures. However, we also observed greater survival after extreme heat in populations from colder latitudes than in their warmer‐latitude counterparts, demonstrating a pattern known as countergradient variation.

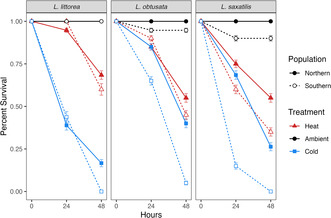

## 1 Introduction

Predicting species vulnerability to climate change, as well as the vulnerability of populations within species, is a fundamental goal in biodiversity conservation. Local adaptation plays a valuable role in mediating species' responses to climate change and refers to the process by which a given population evolves to be better adapted to its environment than populations of the same species from other environments (Razgour et al. 2019; Thompson and Fronhofer 2019). More often than not, however, climate change projections exclude factors like local adaptation, largely due to a lack of necessary data on underlying mechanisms such as population differentiation and the impacts of environmental variation (Urban et al. 2016). To offer representative predictions of species' future success, therefore, it is essential that we first gain a stronger knowledge of the extent of vulnerability among natural populations in differing environmental regimes.

One factor contributing to local adaptation is the occurrence of covariance between genetic and environmental effects on phenotypes (Cov_GE_), which can cause genotypes to be distributed non‐randomly across an environmental gradient (Conover and Schultz 1995; Trussell and Etter 2001). Spatial Cov_GE_ may emerge in a pattern that either amplifies environmental variation or dampens it, respectively, maximising or minimising phenotypic variation across environments (Urban et al. 2020). Past studies (Conover, Duffy, and Hice 2009; Hoffmann and Sgrò 2011; Savolainen, Lascoux, and Merilä 2013; Hu et al. 2020) have demonstrated that organisms' specific genotypic and environmental relationships may form the basis for their differing responses to environmental gradients; thus, as the magnitude and direction of Cov_GE_ can play a fundamental role in determining organisms' responses to changing or novel environments, an understanding of these interactions is essential to robustly predicting species outcomes under climate change.

When Cov_GE_ is negative, a system demonstrates countergradient variation, in which the environmental influence on the phenotype is opposed by the genetic influence, thereby minimising phenotypic variation from the environment to environment. As such, countergradient variation evolves counterintuitive patterns in nature, such as the consistently faster growth rates of northern (cold‐water) Atlantic silversides than those of their southern (warm‐water) counterparts (Conover and Schultz 1995). Countergradient variation is predicted to be common in nature, having already been observed in many species, with many more likely candidates that have yet to be examined (Conover, Duffy, and Hice 2009). In particular, multi‐species comparisons of countergradient variation are lacking, although they are highly relevant to understanding local adaptation in the context of environmental change.

Temperature is frequently an important factor in the occurrence of local adaptation and plays a driving role in the biogeography of ectotherms (Pörtner 2002). As such, changing temperatures concomitant with global climate change have the potential to substantially alter ectotherms' distribution patterns, especially those in marine ecosystems (Fields et al. 1993). Notably, ectothermic inhabitants of the rocky shore intertidal zone must also cope with their habitat's extreme temperature fluctuations, which vary geographically (Helmuth et al. 2002; Sunday, Bates, and Dulvy 2011). Furthermore, the body temperatures of marine ectotherms are closer to their upper thermal limits than those of terrestrial ectotherms (Pinsky et al. 2019). As the threat presented by climate change often correlates positively with an organism's proximity to its upper thermal limit (Diamond et al. 2012; Hamblin et al. 2017), and estimates of organismal thermal tolerance have been shown to correlate closely with mortality from chronic heat stress (Cicchino, Ghalambor, and Funk 2023), heat tolerance is therefore an important metric for understanding populations' ability to persist under consistently warming temperatures. Moreover, the general pattern of increasing temperatures under climate change is accompanied by the occurrence of opposite extremes, including extremely cold winter temperature events (Firth, Knights, and Bell 2011; Wethey et al. 2011), which therefore necessitates an understanding of species' cold tolerance as well.

For marine intertidal species, the environmental conditions influencing species' thermal tolerance are a product of the organism's geospatial location, which can include vertical and latitudinal positions. Species' vertical distribution in the intertidal zone contributes to their physiological tolerance of thermal extremes, as higher zones are exposed to terrestrial conditions for a greater portion of each tidal cycle and thereby experience greater fluctuations in temperature (Murphy 1979; Stillman 2002; Stickle, Lindeberg, and Rice 2015). Further, research has found a negative relationship between increasing latitude and downward shifts in both upper and lower thermal limits at the species level (Sunday, Bates, and Dulvy 2011) in addition to further divergence at the population level (Sasaki et al. 2022). This contrasts with the trend of increasingly variable temperatures as latitude increases (Stevens 1989; Gaston and Chown 1999), as unlike in terrestrial ectotherms, the ranges of thermal tolerance for higher‐latitude marine ectotherms appear not to widen along with their habitats' greater extents of temperature variability (Sunday et al. 2019). Previous studies comparing related species of marine invertebrates have demonstrated latitudinal variation in thermal tolerance (Sinclair et al. 2004; Dennis, Loomis, and Hellberg 2014), including several studies that have explored divergence among populations within a species (Kuo and Sanford 2009; Brahim and Marshall 2020; Dwane et al. 2021, 2023; Sasaki et al. 2022). However, these studies have typically focused specifically on either heat tolerance or cold tolerance, highlighting the need for more studies that compare the responses of a consistent set of species and populations to both hot and cold extremes.

**FIGURE 1:**
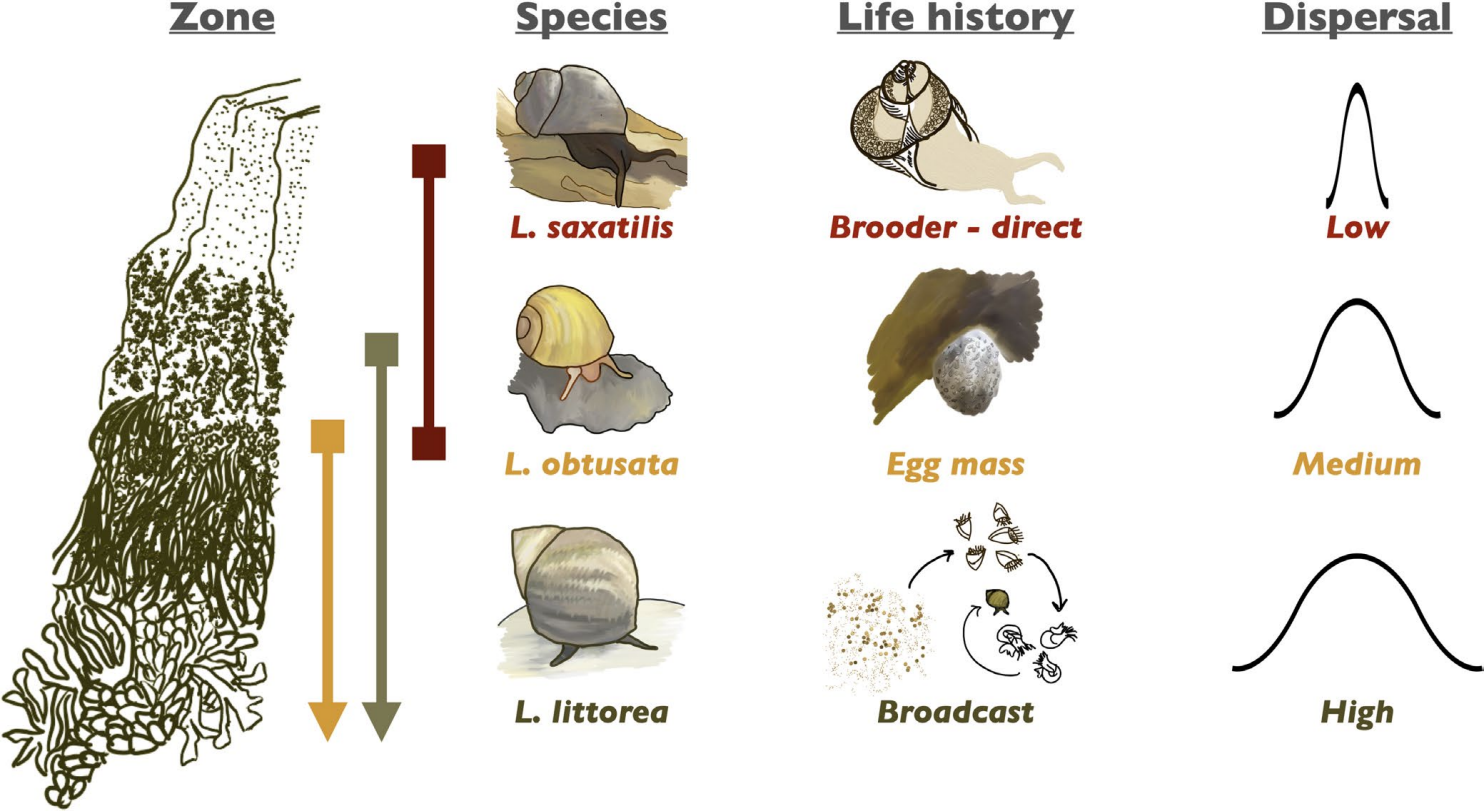
Intertidal zone presence, life history, and dispersal potential of *Littorina saxatilis* , *L. obtusata* , and *L. littorea* .

*Littorina* species are among the most abundant marine gastropods worldwide and inhabit the intertidal zone of rocky shores in northern temperate waters (Reid 1989). Of the three species in this study, *L. obtusata* is most abundant in lower tidal zones, *L. littorea* is typically present in low and mid‐tidal zones, including tide pools, and *L. saxatilis* primarily occurs in the upper intertidal, thus experiencing the greatest amount of air exposure during a single tidal cycle (Yamada and Mansour 1987; Kozminsky 2013) (Figure 1). *L. saxatilis* notably exhibits behavioural adaptations to avoid desiccation during low tide, principally the tight closure of their operculum in a “standing” posture and the formation of aggregations, that together reduce evaporative water loss (Newell 1979; Atkinson and Newbury 1984). However, these behaviours have limited utility in relieving heat stress: the standing posture has been shown to lower body temperatures by an average of only 1°C–2°C, and aggregation behaviour appears not to provide a thermoregulatory benefit (Chapperon, Studerus, and Clavier 2017).

Additionally, the population connectivity of *Littorina* species is impacted by divergent larval development strategies. *L. littorea* reproduces through broadcast spawning with pelagic larvae that undergo long‐range dispersal, while in contrast, *L. obtusata* forgoes a dispersive larval period and instead directly develops from eggs into crawling juveniles. *L. saxatilis* also undergoes direct development, but rather than laying eggs, it is the sole littorinid to brood and birth live young (Reid 1996; Johannesson 2003) (Figure 1). According to population genetic theory, dispersal ability generally relates inversely to population structure (Bohonak 1999), suggesting that these littorinids likely exhibit differing levels of inter‐population variation between species.

Here, we take advantage of a system of sympatric intertidal snails with diverse ecological and life‐history traits to test hypotheses about the effects of thermal extremes on mortality among species and among populations within species. Among species, we predicted that the higher a species lives in the intertidal zone, and therefore the greater its exposure to thermal extremes during the tidal cycle, the lower its demonstrated mortality would be at both thermal extremes. Within species, we predicted that the level of divergence in thermal tolerance among populations would be negatively associated with dispersal potential. As such, this study expands our understanding of the species‐ and population‐level variation in thermal tolerance among marine invertebrate species and provides key information about the geographic dynamics that may play an important role in the persistence of marine ectotherms under climate change scenarios.

## 2 Methods

### 2.1 Sample Collection

**FIGURE 2:**
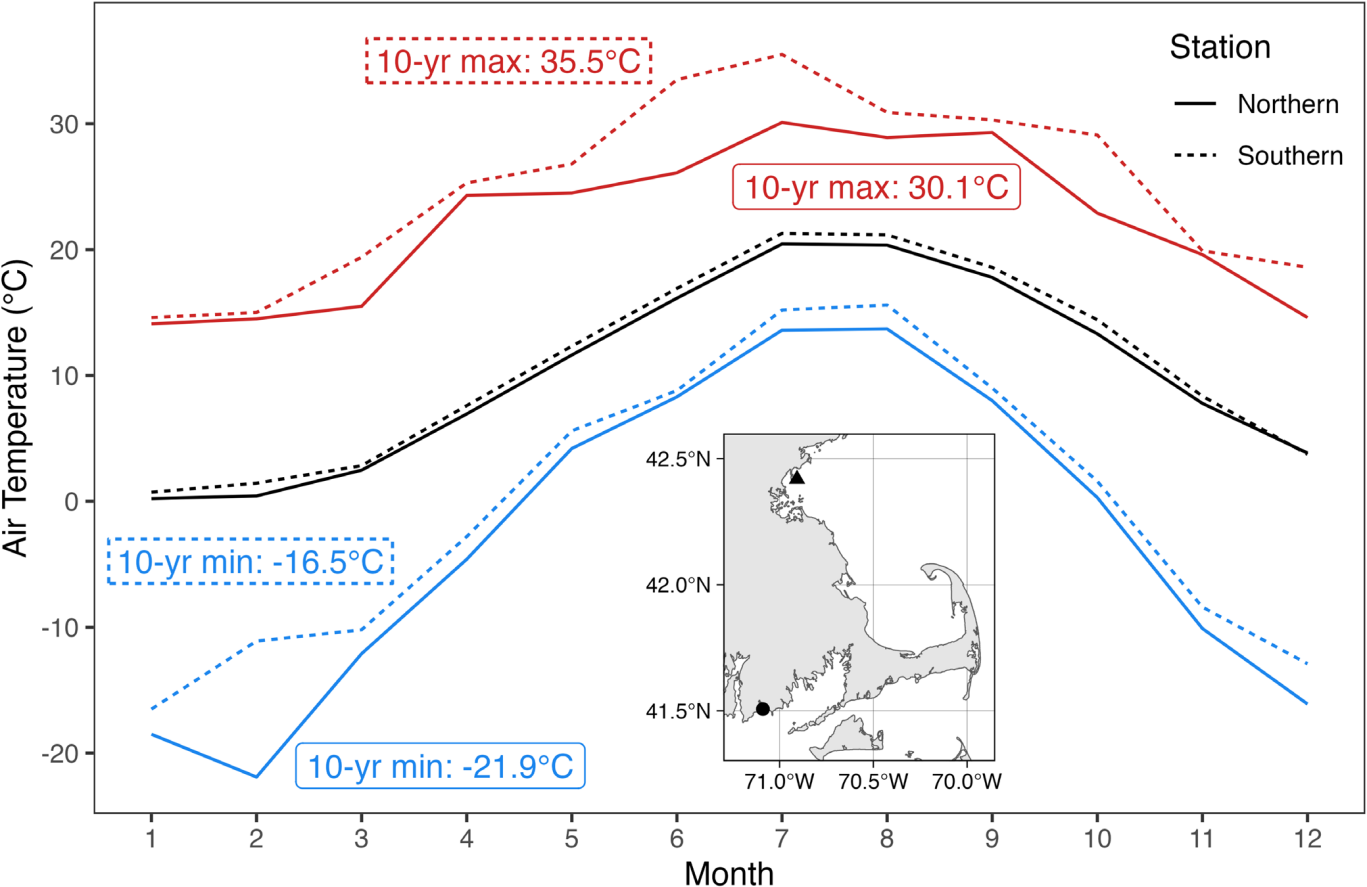
Ten‐year (2010–2019) monthly maximum (red), minimum (blue), and mean (black) air temperatures for specimen collection sites. Data sourced from NOAA's National Data Buoy Center (NDBC). Solid lines represent data from northern station BHBM3 (42.355° N, 71.05° W), and dashed lines represent southern station NWPR1 (41.504° N, 71.326° W). Inset: Locations of northern (triangle) and southern (circle) specimen collection sites relative to Cape Cod, MA.

Individuals of *L. littorea* , *L. obtusata* , and *L. saxatilis* were collected in late June to early July of 2019 at two rocky shore sites north and south of the biogeographic break at Cape Cod (Allee 1923; Pappalardo et al. 2015). In the week prior to collection, mean air temperatures between sites were within 2.5°C of one another (National Data Buoy Center 2019a, 2019b). The first site, in Nahant, MA on Massachusetts Bay (42.4194° N 70.9069° W), USA, was defined as the “northern” population (Figure 2 inset, triangle), and the second, in Acoaxet, MA on Rhode Island Sound (41.5069° N 71.0889° W), USA, was defined as the “southern” population (Figure 2 inset, circle). Over the 10 years before sampling, the northern locality's maximum and minimum monthly mean temperatures were each 5.4°C lower than in the southern locality (Figure 2), representing the likely variation in thermal extremes between sites. Notably, existing genetic work on *L. saxatilis* indicates that populations of *L. saxatilis* south of Cape Cod—the origin of our southern population—represent a single lineage and have very low haplotype diversity, while a second haplogroup is also present among those in the southern Gulf of Maine (i.e., north of Cape Cod) – the origin of our northern population (Doellman et al. 2011; Panova et al. 2011). While similar work has not been conducted on *L. obtusata* , it is possible that similar patterns may exist between the northern and southern sides of Cape Cod due to *L. obtusata* 's somewhat restricted dispersal.

The snails were placed in prelabeled, perforated specimen containers (120 mL) with a layer of aquarium gravel and fresh thalli of brown algae ( *Fucus spiralis* and *Ascophyllum nodosum* ), and only individuals without barnacles or other epizoic organisms were collected to avoid external stressors on the organisms.

### 2.2 Acclimation

**FIGURE 3:**
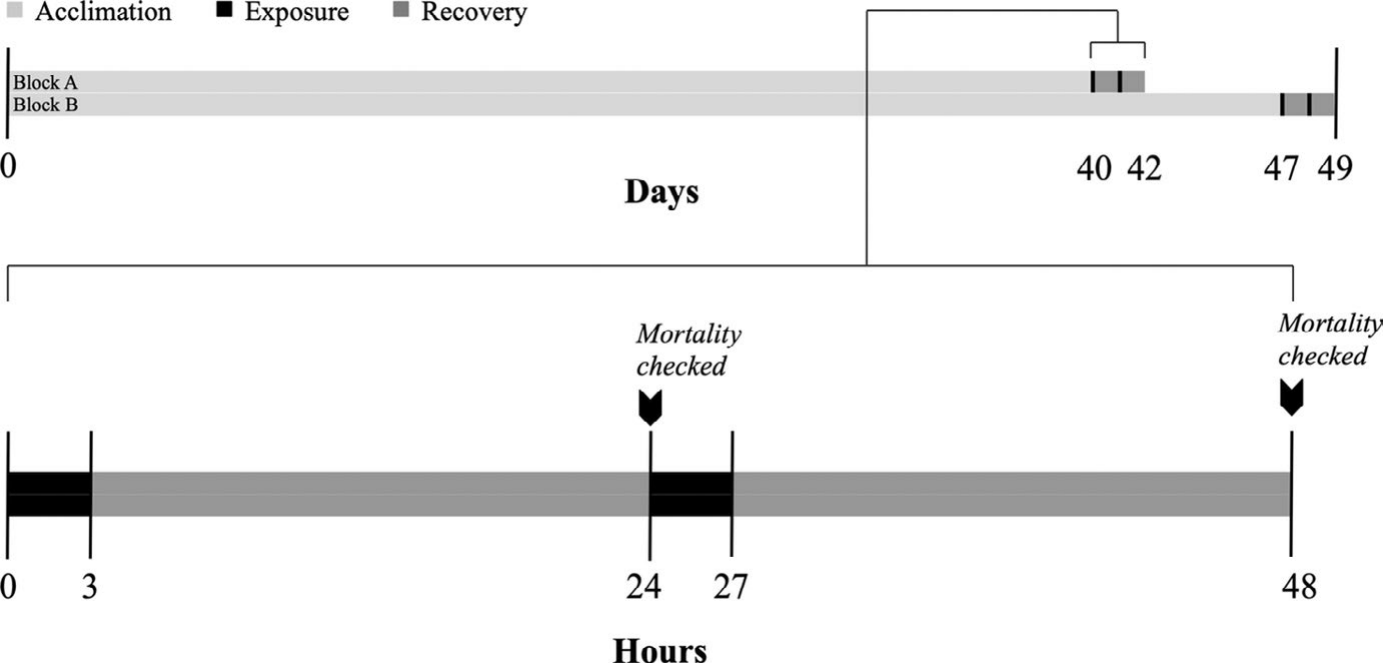
Timeline of acclimation, exposure, and recovery periods.

Our experiment was set up in a block design with the 120‐mL specimen containers placed into seatables (blocks) for acclimation in June 2019. Snails were acclimated for 40–47 days (Figure 3; Block 1: 40 days, Block 2: 47 days) with a twelve‐hour light cycle and at ambient air temperature (20°C–21°C) to reduce the possible influence of phenotypic plasticity on results. The seatables were gradually drained each morning and refilled with filtered seawater from Massachusetts Bay in the afternoon to simulate the tidal cycle. When full, the seatables were under constant flow‐through, and the double layer of specimen containers was completely submerged in seawater. The flow‐through seawater temperature averaged 17.8°C ± 0.51°C (mean ± SE). The snails were provided with fresh thalli of *F. spiralis* and *A. nodosum* at roughly three‐week intervals, and thalli were cleaned with warm freshwater to remove dirt and clinging animals before being placed in the sample containers.

During acclimation, the wet weight (g) of each snail was measured using a digital balance, and snails exceeding 1 g were excluded from the analyses to minimise the confounding effect of body size.

### 2.3 Thermal Exposure

After a block's acclimation period, a static temperature stress assay (Lutterschmidt and Hutchison 1997) was conducted in which snails were exposed to one of three thermal treatments on a 48‐h exposure‐recovery timeline: extreme heat (mean ± SE: 44.75°C ± 1.11°C, *n* = 4 observations over 2 blocks), extreme cold (mean ± SE: −12.25°C ± 0.25°C), or control (ambient) temperature (mean ± SE: 20°C ± 0.41°C) (Figure 3), with 10 snails per treatment‐by‐population‐by‐species replicate for a total of 180 snails per block.

Since the long‐term persistence of marine intertidal gastropods is more dependent on extreme thermal events than on mean temperatures (Denny et al. 2009; Wethey et al. 2011), the thermal treatment temperatures were determined based on known thermal limits of *Littorina* species snails. The heat shock temperature was set at 45°C based on a study exploring the vulnerability of intertidal snails including *L. brevicula* to heat stress (Dong et al. 2017) and a study examining the lethal high temperatures for several marine invertebrates including *L. littorea* (Fraenkel 1960). The heat exposure was implemented in a wooden box with four 70‐watt PAR38 flood light bulbs, which were turned on 30 min before exposure to allow the box to reach temperature. Snails, in specimen containers holding rocks and fresh algae, were placed in the heat treatment with their opaque container lids in place to shield organisms from direct light exposure. The cold shock temperature was set at −12°C in accordance with studies investigating freezing‐induced muscle injury in *L. littorea*, *L. obtusata* , and *L. saxatilis* and the roles of freezing temperature and duration on *L. littorea* mortality (Murphy 1979; Murphy and Johnson 1980). The cold exposure was implemented by placing snails, likewise in lidded specimen cups containing rocks and fresh algae, in a −12°C freezer. In the control treatment, snails were placed in a dry sea table at ambient air temperature. In all treatments, a temperature probe was inserted through the lid of an additional specimen container included in each treatment to measure the temperature inside.

At the beginning of the 48‐h experimental timeline, snails were exposed to their respective treatments for 3 h and then returned to the seatables, mimicking an open‐air exposure around the low tide. Mortality was checked for all snails 21 h post‐exposure, with survival recorded if a snail (1) was attached by its foot to the container wall, base, or lid; (2) responded by moving or opening its operculum when submerged in water; or (3) responded to a physical stimulus. All surviving snails were exposed once again to their respective treatments for another 3 h, after which they were returned to the seatables; after another 21 h, mortality was again checked and recorded.

### 2.4 Statistical Analysis

To test for the effects of species, population, and thermal treatment on snail mortality, we created a generalised linear mixed‐effects model using the package *lme4* (Bates et al. 2015) with species ( *L. littorea* , *L. obtusata* , or *L. saxatilis* ), population (northern or southern), treatment (heat exposure, cold exposure, or control), and block (A or B) all treated as fixed effects. We included block as a fixed effect instead of a random effect because two levels are too few to accurately estimate the variance of random effects (Crawley 2002). The data demonstrated overdispersion with unequal variance across data points. We, therefore, used a bias‐reduced generalised linear model with the R package *brglm2* (Kosmidis and Firth 2021; Kosmidis et al. 2021) and modelled the data as a quasibinomial distribution with the logit link function. The bias‐reduced model was able to improve estimates of standard error compared to a traditional generalised linear model's high standard error values for all coefficients, due to the control treatment's near‐100% survival.

We then proceeded with model selection by removing first the four‐way interaction, and then subsequent three‐way and two‐way interactions in a model selection approach based on Akaike information criterion (AIC) values. Following the model selection process, we calculated specific contrasts to examine our a priori hypotheses about species and population effects on survival within the best model. We then corrected for multiple comparisons (18 tests conducted) using the Benjamini and Hochberg (1995).

### 2.5 Cov_GE_ Calculations

Cov_GE_ estimates were calculated using the framework laid out in Albecker, Trussell, and Lotterhos (2022). The phenotypic data (proportion of snails surviving) were standardised by subtracting the overall mean phenotype from each phenotypic data point and then dividing by the standard deviation of group means, where the “group” referred to the genotype (e.g., each species and location group) and experimental environment (thermal exposure treatment) pair. The genotypic mean phenotype was determined by calculating the mean phenotype for each genotype across environments, and the experimental environment mean phenotype was calculated as the mean phenotype for each environment across genotypes. An ANOVA was used to extract estimated marginal means for each genotype and environmental mean. Finally, 95% confidence intervals were generated using bootstrapping with 999 runs, and hypothesis testing was conducted using permutation to determine statistical significance testing the null hypothesis that Cov_GE_ = 0, with results interpreted as significant if the 95% confidence interval did not include zero.

## 3 Results

After conducting our model selection process using AIC comparisons, we determined the best model to be a bias‐reduced generalised linear model that included the main effects of species, population, treatment, and block, along with the interactions of species: population, species: treatment, population: treatment, and treatment: block. We conducted a series of a priori contrasts to help interpret the multiple two‐way interactions (Table 1).

**TABLE 1:** Effect sizes and significance values for all a priori contrasts.

Question	Contrast (group 1—group 2)	Direction of effect (group 1—group 2)	*P*	*P*_*corrected*_
In control conditions, is there a pairwise species difference in logit‐transformed mortality?	*littorea*—*obtusata*	NS	0.33	0.41
	*littorea*—*saxatilis*	NS	0.28	0.39
	*obtusata*—*saxatilis*	NS	0.88	0.88
In control conditions, is there a pairwise population difference in logit‐transformed mortality?	*littorea* N—*littorea* S	NS	0.23	0.35
	*obtusata* N—*obtusata* S	NS	0.09	0.17
	*saxatilis* N—*saxatilis* S	NS	0.02	0.05
In control conditions, are there overall population differences in logit‐transformed mortality?	N—S	NS	0.07	0.15
In heat conditions, is there a pairwise species difference in logit‐transformed mortality?	*littorea*—*obtusata*	NS	0.05	0.12
	*littorea*—*saxatilis*	+	5 × 10^−4^	0.003
	*obtusata*—*saxatilis*	NS	0.08	0.15
In heat conditions, is there a pairwise population difference in logit‐transformed mortality?	*littorea* N—*littorea* S	NS	0.47	0.54
	*obtusata* N—*obtusata* S	NS	0.64	0.71
	*saxatilis* N—*saxatilis* S	+	0.009	0.03
In heat conditions, are there overall population differences in logit‐transformed mortality?	N—S	NS	0.32	0.41
In cold conditions, is there a pairwise species difference in logit‐transformed mortality?	*littorea*—*obtusata*	−	0.004	0.02
	*littorea*—*saxatilis*	NS	0.87	0.88
	*obtusata*—*saxatilis*	+	0.002	0.01
In cold conditions, is there a pairwise population difference in logit‐transformed mortality?	*littorea* N—*littorea* S	NS	0.21	0.35
	*obtusata* N—*obtusata* S	+	0.005	0.02
	*saxatilis* N—*saxatilis* S	+	1 × 10^−5^	2 × 10^−4^
In cold conditions, are there overall population differences in logit‐transformed mortality?	N—S	+	8 × 10^−5^	7 × 10^−4^

**FIGURE 4:**
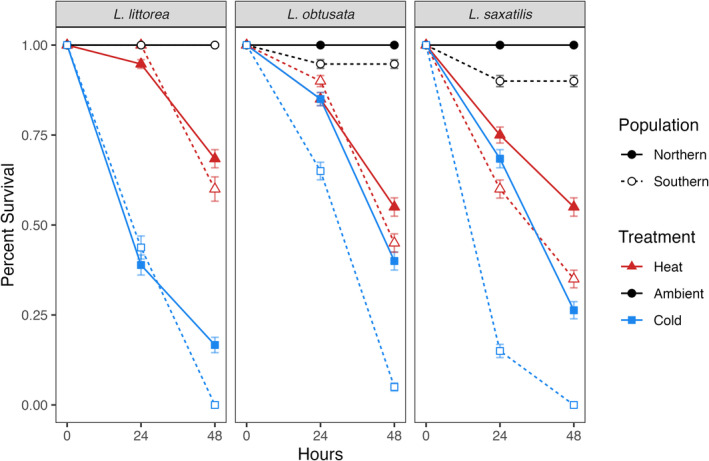
Post‐exposure survival of *Littorina* snails by population and treatment. Error bars represent mean ± SE.

In the control conditions, the survival of all species was almost 100%, and there were no significant differences across species, across populations within a species, or across populations overall (Figure 4). Meanwhile, the snails' heat and cold tolerances were similar across species with several notable contrasts. Following heat exposure, the higher survival of *L. littorea* compared to *L. saxatilis* represented a significant difference (*p* < 0.001) (Figure 4, red lines). Following cold exposure, the higher survival of *L. littorea* compared to *L. obtusata* and of *L. obtusata* compared to *L. saxatilis* likewise represented significant differences (*p* < 0.01) (Figure 4, blue lines).

Examining the populations within each species, the northern and southern populations of *L. littorea* were not significantly different in either the hot or cold treatments (Figure 4, left panel). The northern and southern populations of *L. obtusata* similarly did not demonstrate significantly different mortality following heat exposure, but following cold exposure, the northern population fared significantly better than the southern population (*p* < 0.01) (Figure 4, centre panel). Meanwhile, the northern population of *L. saxatilis* exhibited significantly higher survival after both heat and cold exposure than their southern counterparts (*p* < 0.01, *p* = 1 × 10^−5^, respectively) (Figure 4, right panel).

We also observed a main effect of population in the cold exposure, which must be interpreted in light of the interaction. Across populations, snails did not show significant differences in heat tolerance between the northern and southern groups; however, the southern populations' overall survival following cold exposure was significantly lower than the northern populations' survival (*p <* 1 × 10^−4^), a result that was driven in large part by the significant differentiation between the two populations of *L. obtusata* and *L. saxatilis* .

### 3.1 Patterns of Countergradient Variation

**FIGURE 5:**
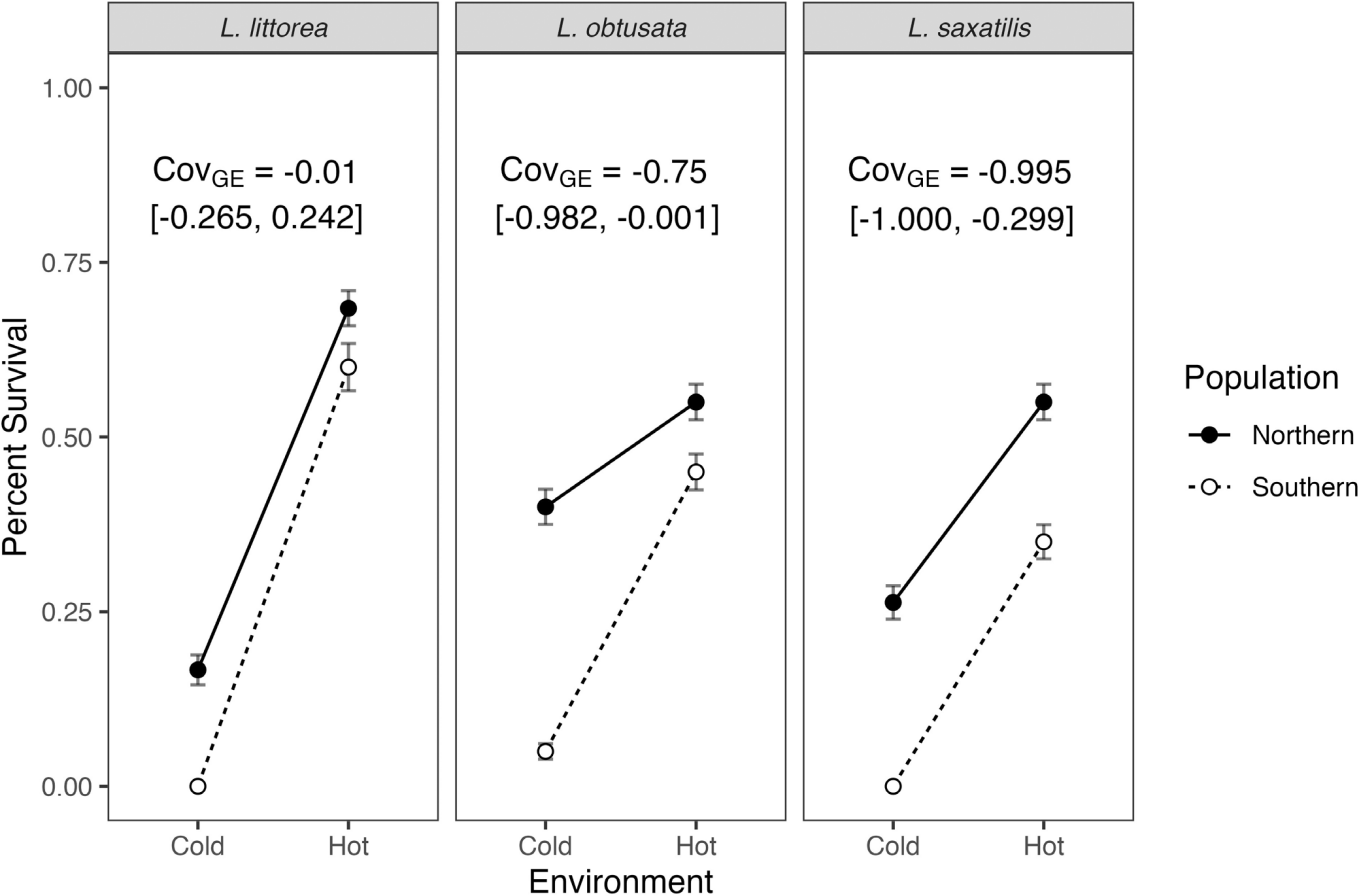
Survival of snails following two consecutive exposures (48‐h time point). Environments refer to extreme heat and cold treatments. Error bars represent the mean ± SE.

Across all species studied, the northern populations of snails exhibited higher survival than their respective southern populations in both the cold and heat exposures, indicating a pattern of countergradient variation among these species (Figure 5). Of the three species, *L. saxatilis* exhibited the strongest countergradient variation in the proportion of snails surviving (Cov_GE_ = −0.995 (95% CI [−1.000, −0.299])) (Figure 5, right panel). *L. obtusata* showed slightly weaker but still distinct evidence of countergradient variation (Cov_GE_ = −0.750 (95% CI [−0.982, −0.001])) (Figure 5, centre panel), while *L. littorea* exhibited no gradient variation (Cov_GE_ = −0.010 (95% CI [−0.265, 0.242])) (Figure 5, left panel).

## 4 Discussion

Here we took a comparative approach to investigating species‐ and population‐level variation in organisms' tolerance of high and low thermal extremes. Counter to our initial hypothesis, the vertical distribution of the three species in the intertidal zone showed only a limited correlation with their tolerance of upper and lower thermal extremes, with three specific pairings presenting as significant. In two of these cases, the species exhibiting significantly lower survival was *L. saxatilis* , which was contrary to the expectation that it should have the greatest tolerance of extremes due to the species' greater air exposure during a tidal cycle (Table 2). At the same time, this study's focus on mature individuals precluded observation of the potential variation among species due to influences on their earlier life stages. As the three species reproduce through three distinct modes, exposure to thermal extremes might induce differential impacts during their earlier stages corresponding to the vulnerability of their respective eggs, embryos, or veligers (larvae). *L. obtusata* larvae, for example, have been found to become more physiologically sensitive as they develop (Bitterli, Rundle, and Spicer 2012), and populations of *L. saxatilis* in the eastern north Atlantic displayed increased inter‐ecotype divergence in heat tolerance as they progressed through their life history stages (Dwane et al. 2021). Further, if these patterns of physiology and development persist once snails are adults but continue to age, then potential variability between the age structures of the focal *Littorina* populations could complicate an interpretation of the inter‐species variability.

**TABLE 2:** Comparison of hypotheses and results for the respective species' levels of thermal tolerance and population divergence.

Species	Mortality in cold shock		Mortality in heat shock		Population divergence	
	Hypothesis	Result	Hypothesis	Result	Hypothesis	Result
*L. littorea* (middle intertidal)	Medium	High	Medium	Low	Lowest	Lowest
*L. obtusata* (low intertidal)	High	Medium	High	Medium	Middle	Middle
*L. saxatilis* (high intertidal)	Low	High	Low	High	Highest	Highest

Population‐level divergence within species was largely consistent with our expectations based on each species' potential for larval dispersal. As a broadcast spawner, *L. littorea* 's lack of population variance in survival rates aligns with their pelagic larval stage in accordance with prior research demonstrating that *Littorina* species with planktonic larvae often show less genetic structure than direct‐developing species (Kyle and Boulding 2000; Blakeslee et al. 2021), in addition to a minimal latitudinal cline in thermal tolerance (Lee and Boulding 2010). Thus, this dispersal ability allows for panmictic populations that demonstrate minimal local adaptation, at least on the scale of the latitudinal difference in this study. Interestingly, Chiba et al. (2016) found that *L. brevicula*, another broadcast‐spawning *Littorina* species, exhibited variation in cold tolerance among populations that experienced latitudinally different cold extremes, suggesting that even species with pelagic larvae may have the capacity to exhibit differentiation among populations distributed across a broader latitudinal gradient. Notably, though, these population localities covered a wider range of latitudes (31° N–44° N) than those included in this study; thus, Chiba et al.'s proposed mechanism of variation, wherein individuals randomly recruit to different locations and those with locally adaptive traits survive (intragenerational selection; Kurihara et al. 2006), might still be at play among populations of *L. littorea* on a broader geographic scale. As such, future work would do well to examine populations from multiple localities across a broader latitudinal gradient, including population replicates.

The relative lack of differentiation between populations of *L. obtusata* suggests that their populations may experience an intermediate degree of admixture, likely because both egg masses and adult snails on macroalgae are liable to raft longer distances should the algae become detached (Reid 1996). This outcome agrees with previous findings that populations of *L. obtusata* exhibit low but nonetheless discernable genetic variation compared to either *L. littorea* or *L. saxatilis* (Berger 1973; Wares and Cunningham 2001; Schmidt et al. 2007). The much stronger divergence observed in this study between northern and southern populations of *L. saxatilis* fits with their contrastingly low dispersal, with live birth of offspring facilitating local adaptation of individual populations to their local conditions. Further, the direction of population‐level variation in northern and southern *L. saxatilis* snails' cold tolerance was consistent with data indicating that organisms from higher latitudes are often better adapted to extreme cold. The lack of a significant difference in northern and southern *L. saxatilis* survival following heat exposure, however, suggests that the differential in maximum temperatures between the two sampling locations may not be substantial enough to have resulted in differing thermal tolerance. Alternatively, the patterns observed may be influenced by the variable genetic lineages underlying each population: this level of genetic divergence may translate to contrasting degrees of thermal adaptation among individuals within the single focal population, thereby confounding a population‐level assessment of tolerance. Relatedly, Dwane et al. (2021) measured different degrees of thermal tolerance between two *L. saxatilis* ecotypes, and while care was taken in this study to collect specimens from a consistent region of the intertidal zone, the potential inclusion of differently adapted ecotypes could likewise confound patterns of inter‐population variation.

It is worth noting as well that the ability to directly compare these species' divergence due to dispersal potential could be inhibited by microhabitat structuring that differs among localities (Lathlean, Ayre, and Minchinton 2014), for example offering more or less availability of thermal refugia that can allow intertidal gastropods to somewhat reduce the costs of exposure to extreme temperatures (Reid and Harley 2021; Dong et al. 2022). A differential in food availability and/or quality between localities could similarly influence the observed inter‐population results. Moreover, as several studies have demonstrated that air temperature alone may not fully predict intertidal organisms' body temperatures (Gilman, Wethey, and Helmuth 2006; Helmuth et al. 2006, 2011), it is also possible that additional factors such as regional tidal patterns or organisms' body temperature sensitivity may vary between the two population sites, offsetting the difference in temperatures.

Overall, the higher survival of both *L. littorea* populations following heat exposure suggests that this species has the potential to respond better to increasing temperatures across populations, representing a likely advantage of high dispersal and probable higher gene flow. Meanwhile, *L. saxatilis* appears to have the potential for higher survival only in its northern population, which presents a possible downside or constraint to the local adaptation demonstrated by species with lower dispersal. Valuably, however, the countergradient variation observed among these species suggests that northern populations' adaptation to an overall cooler climate does not seem to leave them at a disadvantage in tolerating increasingly extreme high temperatures. In fact, they may even have an advantage: counterintuitively, other studies have shown that some of the most heat‐adapted marine organisms may be most at risk under rising temperatures due to constraints on their ability to tolerate increased heat (Pinsky et al. 2019; Dong et al. 2022), while organisms at higher latitudes have a greater capacity to acclimatise.

Through these results, this study highlights an excellent system to be used in further exploration of pressing questions around species' potential for adaptation under climate change, and in pursuing parallel lines of inquiry such as analyses of population differentiation at the genetic level (e.g., Blakeslee et al. 2021). Subsequent work would do well to evaluate the impacts of heat or cold exposure at more than two temperatures: although the temperatures used in this study were opposite extremes, the incorporation of a thermal performance curve would provide a more nuanced picture of the species' thermal tolerance. Additionally, as survival alone provides limited insight into organismal fitness, future studies would benefit from the use of continuous physiological metrics such as cardiac activity or activity coefficients. Dwane et al. (2023) provide an excellent model of this style of experimental design applied to *L. saxatilis* , as do Reid and Harley (2021) in their study on *L. scutulata* . Future work could also consider the incorporation of additional successive exposures to extreme temperatures beyond the two 24‐h cycles implemented here: in the natural world, organisms are threatened not only by individually stressful thermal events but also by the cumulative impact of recurring thermal stress (Rezende et al. 2020), so such a design could offer added perspective on the extent of focal organisms' thermal tolerance.

### 4.1 Patterns of Countergradient Variation

Multiple‐species comparisons of countergradient variation provide valuable insight into questions about local adaptation and environmental change. As such, this study broadens our basis by which to understand intra‐species responses to the changing climate. While northern populations might be expected to have greater cold tolerance due to their native environment, the greater tolerance of northern populations to heat conditions presented a distinctive pattern of countergradient variation. This outcome concurred with the findings of Dwane et al. (2023) that the thermal optima and upper thermal limits of focal *L. saxatilis* populations increased with latitude to their highest values in the northernmost population. Countergradient variation has also previously been observed in the growth of *L. obtusata* snails in response to water velocity, although not to thermal conditions, setting a precedent that gradient variation is possible in the species (Trussell 2002). Further, as temperatures tend to vary more dramatically with increasing latitude, it is reasonable that populations at higher latitudes may undergo adaptation to exhibit greater tolerance for both extreme hot and extreme cold temperatures (although prior studies have shown otherwise, e.g. Sunday et al. 2019). There is no theory yet that describes why countergradient variation evolves in some species but not in others; nonetheless, understanding these interactions between genotype and environment is critical to improving our accuracy in predicting species responses to climate change.

## Author Contributions

**Ffion Dylan Titmuss:** data curation (lead), formal analysis (equal), investigation (lead), methodology (equal), writing – original draft (lead), writing – review and editing (equal). **Molly A. Albecker:** formal analysis (equal), writing – review and editing (equal). **Katie E. Lotterhos:** conceptualization (lead), formal analysis (equal), funding acquisition (lead), methodology (equal), project administration (lead), writing – review and editing (equal).

## Conflicts of Interest

The authors declare no conflicts of interest.

### Open Research Badges

This article has earned an Open Data badge for making publicly available the digitally‐shareable data necessary to reproduce the reported results. The data is available at https://doi.org/10.5061/dryad.ht76hdrnx.

## Data Availability

The data supporting this study's findings are available in Dryad at https://doi.org/10.5061/dryad.ht76hdrnx. The code used for analyses is available at https://doi.org/10.5281/zenodo.13738093.

## References

[ece370926-bib-0001] Albecker, M. A. , G. C. Trussell , and K. E. Lotterhos . 2022. “A Novel Analytical Framework to Quantify Co‐Gradient and Countergradient Variation.” Ecology Letters 25: 1521–1533. 10.1111/ele.14020. 35545439

[ece370926-bib-0002] Allee, W. C. 1923. “Studies in Marine Ecology: The Effect of Temperature in Limiting the Geographical Range of Invertebrates of the Woods Hole Littoral.” Ecology 4: 341–354. 10.2307/1929181.

[ece370926-bib-0003] Atkinson, W. D. , and S. F. Newbury . 1984. “The Adaptations of the Rough Winkle, *Littorina rudis*, to Desiccation and to Dislodgement by Wind and Waves.” Journal of Animal Ecology 53: 93–105. 10.2307/4344.

[ece370926-bib-0004] Bates, D. , M. Mächler , B. Bolker , and S. Walker . 2015. “Fitting Linear Mixed‐Effects Models Using Lme4.” Journal of Statistical Software 67: 1–48. 10.18637/jss.v067.i01.

[ece370926-bib-0005] Benjamini, Y. , and Y. Hochberg . 1995. “Controlling the False Discovery Rate: A Practical and Powerful Approach to Multiple Testing.” Journal of the Royal Statistical Society, Series B 57: 289–300.

[ece370926-bib-0006] Berger, E. M. 1973. “Gene‐Enzyme Variation in Three Sympatric Species of Littorina.” Biological Bulletin 145: 83–90. 10.2307/1540349.

[ece370926-bib-0007] Bitterli, T. S. , S. D. Rundle , and J. I. Spicer . 2012. “Development of Cardiovascular Function in the Marine Gastropod *Littorina Obtusata* (Linnaeus).” Journal of Experimental Biology 215: 2327–2333. 10.1242/jeb.067967. 22675194

[ece370926-bib-0008] Blakeslee, A. M. H. , A. W. Miller , G. M. Ruiz , K. Johannesson , C. André , and M. Panova . 2021. “Population Structure and Phylogeography of Two North Atlantic Littorina Species With Contrasting Larval Development.” Marine Biology 168: 117. 10.1007/s00227-021-03918-8.

[ece370926-bib-0300] Bohonak, A. J. 1999. “Dispersal, Gene Flow, and Population Structure.” The Quarterly Review of Biology 74: 21–45. 10.1086/392950. 10081813

[ece370926-bib-0009] Brahim, A. , and D. J. Marshall . 2020. “Differences in Heat Tolerance Plasticity Between Supratidal and Intertidal Snails Indicate Complex Responses to Microhabitat Temperature Variation.” Journal of Thermal Biology 91: 102620. 10.1016/j.jtherbio.2020.102620. 32716870

[ece370926-bib-0010] Chapperon, C. , K. Studerus , and J. Clavier . 2017. “Mitigating Thermal Effect of Behaviour and Microhabitat on the Intertidal Snail *Littorina saxatilis* (Olivi) Over Summer.” Journal of Thermal Biology 67: 40–48. 10.1016/j.jtherbio.2017.03.017. 28558936

[ece370926-bib-0011] Chiba, S. , T. Iida , A. Tomioka , N. Azuma , T. Kurihara , and K. Tanaka . 2016. “Population Divergence in Cold Tolerance of the Intertidal Gastropod Littorina Brevicula Explained by Habitat‐Specific Lowest Air Temperature.” Journal of Experimental Marine Biology and Ecology 481: 49–56. 10.1016/j.jembe.2016.04.009.

[ece370926-bib-0012] Cicchino, A. S. , C. K. Ghalambor , and W. C. Funk . 2023. “Linking Critical Thermal Maximum to Mortality From Thermal Stress in a Cold‐Water Frog.” Biology Letters 19: 20230106. 10.1098/rsbl.2023.0106. 37311548 PMC10264101

[ece370926-bib-0013] Conover, D. O. , T. A. Duffy , and L. A. Hice . 2009. “The Covariance Between Genetic and Environmental Influences Across Ecological Gradients.” Annals of the New York Academy of Sciences 1168: 100–129. 10.1111/j.1749-6632.2009.04575.x. 19566705

[ece370926-bib-0014] Conover, D. O. , and E. T. Schultz . 1995. “Phenotypic Similarity and the Evolutionary Significance of Countergradient Variation.” Trends in Ecology & Evolution 10: 248–252. 10.1016/S0169-5347(00)89081-3. 21237029

[ece370926-bib-0015] Crawley, M. J. 2002. Statistical Computing: An Introduction to Data Analysis Using S‐Plus. Chichester, West Sussex, UK: Wiley.

[ece370926-bib-0016] Dennis, A. B. , S. H. Loomis , and M. E. Hellberg . 2014. “Latitudinal Variation of Freeze Tolerance in Intertidal Marine Snails of the Genus Melampus (Gastropoda: Ellobiidae).” Physiological and Biochemical Zoology 87: 517–526. 10.1086/676138. 24940916

[ece370926-bib-0017] Denny, M. W. , L. J. H. Hunt , L. P. Miller , and C. D. G. Harley . 2009. “On the Prediction of Extreme Ecological Events.” Ecological Monographs 79: 397–421. 10.1890/08-0579.1.

[ece370926-bib-0018] Diamond, S. E. , L. M. Nichols , N. McCoy , et al. 2012. “A Physiological Trait‐Based Approach to Predicting the Responses of Species to Experimental Climate Warming.” Ecology 93: 2305–2312. 10.1890/11-2296.1. 23236901

[ece370926-bib-0019] Doellman, M. M. , G. C. Trussell , J. W. Grahame , and S. V. Vollmer . 2011. “Phylogeographic Analysis Reveals a Deep Lineage Split Within North Atlantic *Littorina saxatilis* .” Proceedings of the Royal Society B: Biological Sciences 278: 3175–3183. 10.1098/rspb.2011.0346. PMC3169032 21429920

[ece370926-bib-0020] Dong, Y. , X. Li , F. M. P. Choi , G. A. Williams , G. N. Somero , and B. Helmuth . 2017. “Untangling the Roles of Microclimate, Behaviour and Physiological Polymorphism in Governing Vulnerability of Intertidal Snails to Heat Stress.” Proceedings of the Royal Society B: Biological Sciences 284: 20162367. 10.1098/rspb.2016.2367. PMC5443929 28469014

[ece370926-bib-0021] Dong, Y. , M. Liao , G. Han , and G. N. Somero . 2022. “An Integrated, Multi‐Level Analysis of Thermal Effects on Intertidal Molluscs for Understanding Species Distribution Patterns.” Biological Reviews 97: 554–581. 10.1111/brv.12811. 34713568

[ece370926-bib-0022] Dwane, C. , E. L. Rezende , O. Tills , et al. 2023. “Thermodynamic Effects Drive Countergradient Responses in the Thermal Performance of *Littorina saxatilis* Across Latitude.” Science of the Total Environment 863: 160877. 10.1016/j.scitotenv.2022.160877. 36521622

[ece370926-bib-0023] Dwane, C. , S. D. Rundle , O. Tills , et al. 2021. “Divergence in Thermal Physiology Could Contribute to Vertical Segregation in Intertidal Ecotypes of *Littorina saxatilis* .” Physiological and Biochemical Zoology 94: 353–365. 10.1086/716176. 34431748

[ece370926-bib-0024] Fields, P. A. , J. B. Graham , R. H. Rosenblatt , and G. N. Somero . 1993. “Effects of Expected Global Climate Change on Marine Faunas.” Trends in Ecology & Evolution 8: 361–367. 10.1016/0169-5347(93)90220-J. 21236196

[ece370926-bib-0025] Firth, L. B. , A. M. Knights , and S. S. Bell . 2011. “Air Temperature and Winter Mortality: Implications for the Persistence of the Invasive Mussel, Perna Viridis in the Intertidal Zone of the South‐Eastern United States.” Journal of Experimental Marine Biology and Ecology Global change in marine ecosystems 400: 250–256. 10.1016/j.jembe.2011.02.007.

[ece370926-bib-0026] Fraenkel, G. 1960. “Lethal High Temperatures for Three Marine Invertebrates: *Limulus polyphemus* , Littorina Littorea and *Pagurus longicarpus* .” Oikos 11: 171–182. 10.2307/3564681.

[ece370926-bib-0027] Gaston, K. J. , and S. L. Chown . 1999. “Why Rapoport's Rule Does Not Generalise.” Oikos 84: 309–312. 10.2307/3546727.

[ece370926-bib-0028] Gilman, S. E. , D. S. Wethey , and B. Helmuth . 2006. “Variation in the Sensitivity of Organismal Body Temperature to Climate Change Over Local and Geographic Scales.” Proceedings of the National Academy of Sciences 103: 9560–9565. 10.1073/pnas.0510992103. PMC1480446 16763050

[ece370926-bib-0029] Hamblin, A. L. , E. Youngsteadt , M. M. López‐Uribe , and S. D. Frank . 2017. “Physiological Thermal Limits Predict Differential Responses of Bees to Urban Heat‐Island Effects.” Biology Letters 13: 20170125. 10.1098/rsbl.2017.0125. 28637837 PMC5493736

[ece370926-bib-0030] Helmuth, B. , B. R. Broitman , C. A. Blanchette , et al. 2006. “Mosaic Patterns of Thermal Stress in the Rocky Intertidal Zone: Implications for Climate Change.” Ecological Monographs 76: 461–479. 10.1890/0012-9615(2006)076[0461:MPOTSI]2.0.CO;2.

[ece370926-bib-0031] Helmuth, B. , C. D. G. Harley , P. M. Halpin , M. O'Donnell , G. E. Hofmann , and C. A. Blanchette . 2002. “Climate Change and Latitudinal Patterns of Intertidal Thermal Stress.” Science 298: 1015–1017. 10.1126/science.1076814. 12411702

[ece370926-bib-0301] Helmuth, B. , L. Yamane , S. Lalwani , A. Matzelle , A. Tockstein , and N. Gao . 2011. “Hidden signals of climate change in intertidal ecosystems: What (not) to expect when you are expecting.” Journal of Experimental Marine Biology and Ecology 400: 191–199. 10.1016/j.jembe.2011.02.004.

[ece370926-bib-0032] Hoffmann, A. A. , and C. M. Sgrò . 2011. “Climate Change and Evolutionary Adaptation.” Nature 470: 479–485. 10.1038/nature09670. 21350480

[ece370926-bib-0033] Hu, Z.‐M. , K.‐L. Zhong , F. Weinberger , D.‐L. Duan , S. G. A. Draisma , and E. A. Serrão . 2020. “Linking Ecology to Genetics to Better Understand Adaptation and Evolution: A Review in Marine Macrophytes.” Frontiers in Marine Science 7: 545102. 10.3389/fmars.2020.545102.

[ece370926-bib-0034] Johannesson, K. 2003. “Evolution in Littorina: Ecology Matters.” Journal of Sea Research 49, no. Structuring Factors of Shallow Marine Coastal Communities, Part II: 107–117. 10.1016/S1385-1101(02)00218-6.

[ece370926-bib-0035] Kosmidis, I. , and D. Firth . 2021. “Jeffreys‐Prior Penalty, Finiteness and Shrinkage in Binomial‐Response Generalized Linear Models.” Biometrika 108: 71–82. 10.1093/biomet/asaa052.

[ece370926-bib-0036] Kosmidis, I. , E. C. K. Pagui , K. Konis , and N. Sartori . 2021. “brglm2: Bias Reduction in Generalized Linear Models.”

[ece370926-bib-0037] Kozminsky, E. V. 2013. “Effects of Environmental and Biotic Factors on the Fluctuations of Abundance of *Littorina obtusata* (Gastropoda: Littorinidae).” Hydrobiologia 706: 81–90. 10.1007/s10750-012-1418-0.

[ece370926-bib-0038] Kuo, E. S. L. , and E. Sanford . 2009. “Geographic Variation in the Upper Thermal Limits of an Intertidal Snail: Implications for Climate Envelope Models.” Marine Ecology Progress Series 388: 137–146. 10.3354/meps08102.

[ece370926-bib-0039] Kurihara, T. , M. Shikatani , K. Nakayama , and M. Nishida . 2006. “Proximate Mechanisms Causing Morphological Variation in a Turban Snail Among Different Shores.” Journal of Zoology 23: 999–1008. 10.2108/zsj.23.999. 17189912

[ece370926-bib-0040] Kyle, C. J. , and E. G. Boulding . 2000. “Comparative Population Genetic Structure of Marine Gastropods (Littorina Spp.) With and Without Pelagic Larval Dispersal.” Marine Biology 137: 835–845. 10.1007/s002270000412.

[ece370926-bib-0041] Lathlean, J. A. , D. J. Ayre , and T. E. Minchinton . 2014. “Estimating Latitudinal Variability in Extreme Heat Stress on Rocky Intertidal Shores.” Journal of Biogeography 41: 1478–1491. 10.1111/jbi.12311.

[ece370926-bib-0042] Lee, H. J. , and E. G. Boulding . 2010. “Latitudinal Clines in Body Size, but Not in Thermal Tolerance or Heat‐Shock Cognate 70 (HSC70), in the Highly‐Dispersing Intertidal Gastropod *Littorina keenae* (Gastropoda: Littorinidae).” Biological Journal of the Linnean Society 100: 494–505. 10.1111/j.1095-8312.2010.01450.x.

[ece370926-bib-0043] Lutterschmidt, W. I. , and V. H. Hutchison . 1997. “The Critical Thermal Maximum: History and Critique.” Canadian Journal of Zoology 75: 1561–1574. 10.1139/z97-783.

[ece370926-bib-0044] Murphy, D. J. 1979. “The Relationship Between the Lethal Freezing Temperatures and the Amounts of Ice Formed in the Foot Muscle of Marine Snails (Mollusca: Gastropoda).” Cryobiology 16: 292–300. 10.1016/0011-2240(79)90041-5. 477372

[ece370926-bib-0045] Murphy, D. J. , and L. C. Johnson . 1980. “Physical and Temporal Factors Influencing the Freezing Tolerance of the Marine Snail *littorina littorea* (l.).” Biological Bulletin 158: 220–232. 10.2307/1540932.

[ece370926-bib-0046] National Data Buoy Center, N.O. and A.A . 2019a. “Historical Observations From Buoy 44013 (42.346N 70.651W)–BOSTON 16 NM East of Boston, MA.” https://www.ndbc.noaa.gov/station_history.php?station=44013.

[ece370926-bib-0047] National Data Buoy Center, N.O. and A.A . 2019b. “Historical Observations From Station NWPR1 (41.504N 71.326W)–8452660–Newport, RI.” https://www.ndbc.noaa.gov/station_history.php?station=nwpr1.

[ece370926-bib-0048] Newell, R. C. 1979. Biology of Intertidal Animals. Faversham, UK: Marine Ecological Surveys.

[ece370926-bib-0049] Panova, M. , A. M. H. Blakeslee , A. W. Miller , et al. 2011. “Glacial History of the North Atlantic Marine Snail, *Littorina saxatilis* , Inferred From Distribution of Mitochondrial DNA Lineages.” PLoS One 6: e17511. 10.1371/journal.pone.0017511. 21412417 PMC3055875

[ece370926-bib-0050] Pappalardo, P. , J. M. Pringle , J. P. Wares , and J. E. Byers . 2015. “The Location, Strength, and Mechanisms Behind Marine Biogeographic Boundaries of the East Coast of North America.” Ecography 38: 722–731. 10.1111/ecog.01135.

[ece370926-bib-0051] Pinsky, M. L. , A. M. Eikeset , D. J. McCauley , J. L. Payne , and J. M. Sunday . 2019. “Greater Vulnerability to Warming of Marine Versus Terrestrial Ectotherms.” Nature 569: 108–111. 10.1038/s41586-019-1132-4. 31019302

[ece370926-bib-0052] Pörtner, H. O. 2002. “Climate Variations and the Physiological Basis of Temperature Dependent Biogeography: Systemic to Molecular Hierarchy of Thermal Tolerance in Animals.” Comparative Biochemistry and Physiology Part A: Molecular & Integrative Physiology 132: 739–761. 10.1016/S1095-6433(02)00045-4. 12095860

[ece370926-bib-0053] Razgour, O. , B. Forester , J. B. Taggart , et al. 2019. “Considering Adaptive Genetic Variation in Climate Change Vulnerability Assessment Reduces Species Range Loss Projections.” Proceedings of the National Academy of Sciences 116: 10418–10423. 10.1073/pnas.1820663116. PMC6535011 31061126

[ece370926-bib-0054] Reid, D. G. 1989. “The Comparative Morphology, Phylogeny and Evolution of the Gastropod Family Littorinidae.” Philosophical Transactions of the Royal Society of London. B, Biological Sciences 324: 1–110. 10.1098/rstb.1989.0040.

[ece370926-bib-0055] Reid, D. G. 1996. Systematics and Evolution of Littorina. London, UK: Ray Society.

[ece370926-bib-0056] Reid, H. , and C. Harley . 2021. “Low Temperature Exposure Determines Performance and Thermal Microhabitat Use in an Intertidal Gastropod ( *Littorina scutulata* ) During the Winter.” Marine Ecology Progress Series 660: 105–118. 10.3354/meps13588.

[ece370926-bib-0057] Rezende, E. L. , F. Bozinovic , A. Szilágyi , and M. Santos . 2020. “Predicting Temperature Mortality and Selection in Natural Drosophila Populations.” Science 369: 1242–1245. 10.1126/science.aba9287. 32883867

[ece370926-bib-0058] Sasaki, M. , J. M. Barley , S. Gignoux‐Wolfsohn , et al. 2022. “Greater Evolutionary Divergence of Thermal Limits Within Marine Than Terrestrial Species.” Nature Climate Change 12: 1175–1180. 10.1038/s41558-022-01534-y.

[ece370926-bib-0059] Savolainen, O. , M. Lascoux , and J. Merilä . 2013. “Ecological Genomics of Local Adaptation.” Nature Reviews. Genetics 14: 807–820. 10.1038/nrg3522. 24136507

[ece370926-bib-0060] Schmidt, P. S. , M. Phifer‐Rixey , G. M. Taylor , and J. Christner . 2007. “Genetic Heterogeneity Among Intertidal Habitats in the Flat Periwinkle, *Littorina obtusata* .” Molecular Ecology 16: 2393–2404. 10.1111/j.1365-294X.2007.03323.x. 17561900

[ece370926-bib-0061] Sinclair, B. J. , D. J. Marshall , S. Singh , and S. L. Chown . 2004. “Cold Tolerance of Littorinidae From Southern Africa: Intertidal Snails Are Not Constrained to Freeze Tolerance.” Journal of Comparative Physiology. B 174: 617–624. 10.1007/s00360-004-0451-3. 15517285

[ece370926-bib-0062] Stevens, G. C. 1989. “The Latitudinal Gradient in Geographical Range: How So Many Species Coexist in the Tropics.” American Naturalist 133: 240–256. 10.1086/284913.

[ece370926-bib-0063] Stickle, W. B. , M. Lindeberg , and S. D. Rice . 2015. “Comparative Freeze Tolerance and Physiological Adaptations of Three Species of Vertically Distributed Rocky Intertidal Gastropods From Southeast Alaska.” Journal of Experimental Marine Biology and Ecology 463: 17–21. 10.1016/j.jembe.2014.10.027.

[ece370926-bib-0064] Stillman, J. H. 2002. “Causes and Consequences of Thermal Tolerance Limits in Rocky Intertidal Porcelain Crabs, Genus Petrolisthes.” Integrative and Comparative Biology 42: 790–796. 10.1093/icb/42.4.790. 21708777

[ece370926-bib-0065] Sunday, J. , J. M. Bennett , P. Calosi , et al. 2019. “Thermal Tolerance Patterns Across Latitude and Elevation.” Philosophical Transactions of the Royal Society, B: Biological Sciences 374: 20190036. 10.1098/rstb.2019.0036. PMC6606462 31203755

[ece370926-bib-0066] Sunday, J. M. , A. E. Bates , and N. K. Dulvy . 2011. “Global Analysis of Thermal Tolerance and Latitude in Ectotherms.” Proceedings of the Royal Society B: Biological Sciences 278: 1823–1830. 10.1098/rspb.2010.1295. PMC3097822 21106582

[ece370926-bib-0067] Thompson, P. L. , and E. A. Fronhofer . 2019. “The Conflict Between Adaptation and Dispersal for Maintaining Biodiversity in Changing Environments.” Proceedings of the National Academy of Sciences 116: 21061–21067. 10.1073/pnas.1911796116. PMC6800316 31570612

[ece370926-bib-0068] Trussell, G. C. 2002. “Evidence of Countergradient Variation in the Growth of an Intertidal Snail in Response to Water Velocity.” Marine Ecology Progress Series 243: 123–131. 10.3354/meps243123.

[ece370926-bib-0069] Trussell, G. C. , and R. J. Etter . 2001. “Integrating Genetic and Environmental Forces That Shape the Evolution of Geographic Variation in a Marine Snail.” Genetica 112: 321–337. 10.1023/A:1013364527698. 11838773

[ece370926-bib-0070] Urban, M. C. , G. Bocedi , A. P. Hendry , et al. 2016. “Improving the Forecast for Biodiversity Under Climate Change.” Science 353: aad8466. 10.1126/science.aad8466. 27609898

[ece370926-bib-0071] Urban, M. C. , S. Y. Strauss , F. Pelletier , et al. 2020. “Evolutionary Origins for Ecological Patterns in Space.” Proceedings of the National Academy of Sciences 117: 17482–17490. 10.1073/pnas.1918960117. PMC7395528 32641501

[ece370926-bib-0072] Wares, J. P. , and C. W. Cunningham . 2001. “Phylogeography and Historical Ecology of the North Atlantic Intertidal.” Evolution 55: 2455–2469. 10.1111/j.0014-3820.2001.tb00760.x. 11831661

[ece370926-bib-0073] Wethey, D. S. , S. A. Woodin , T. J. Hilbish , S. J. Jones , F. P. Lima , and P. M. Brannock . 2011. “Response of Intertidal Populations to Climate: Effects of Extreme Events Versus Long Term Change.” Journal of Experimental Marine Biology and Ecology Global change in marine ecosystems 400: 132–144. 10.1016/j.jembe.2011.02.008.

[ece370926-bib-0074] Yamada, S. B. , and R. A. Mansour . 1987. “Growth Inhibition of Native *Littorina saxatilis* (Olivi) by Introduced *L. littorea* (L.).” Journal of Experimental Marine Biology and Ecology 105: 187–196. 10.1016/0022-0981(87)90171-7.

